# Homogenization of initial cell distribution by secondary flow of medium improves cell culture efficiency

**DOI:** 10.1371/journal.pone.0235827

**Published:** 2020-07-15

**Authors:** Yuki Fukuma, Takumi Inui, Chikahiro Imashiro, Yuta Kurashina, Kenjiro Takemura

**Affiliations:** 1 School of Science for Open and Environmental Systems, Graduate School of Science and Technology, Keio University, Yokohama, Kanagawa, Japan; 2 Department of Mechanical Engineering, Faculty of Science and Technology, Keio University, Yokohama, Kanagawa, Japan; 3 Institute of Advanced Biomedical Engineering and Science, Tokyo Women’s Medical University, Tokyo, Japan; 4 Department of Materials Science and Engineering, School of Materials and Chemical Technology, Tokyo institute of Technology, Yokohama, Kanagawa, Japan; Universiti Putra Malaysia, MALAYSIA

## Abstract

Homogenization of the initial cell distribution is essential for effective cell development. However, there are few previous reports on efficient cell seeding methods, even though the initial cell distribution has a large effect on cell proliferation. Dense cell regions have an inverse impact on cell development, known as contact inhibition. In this study, we developed a method to homogenize the cell seeding density using secondary flow, or Ekman transportation, induced by orbital movement of the culture dish. We developed an orbital shaker device that can stir the medium in a 35-mm culture dish by shaking the dish along a circular orbit with 2 mm of eccentricity. The distribution of cells in the culture dish can be controlled by the rotational speed of the orbital shaker, enabling dispersion of the initial cell distribution. The experimental results indicated that the cell density became most homogeneous at 61 rpm. We further evaluated the cell proliferation after homogenization of the initial cell density at 61 rpm. The results revealed 36% higher proliferation for the stirred samples compared with the non-stirred control samples. The present findings indicate that homogenization of the initial cell density by Ekman transportation contributes to the achievement of higher cell proliferation.

## Introduction

Regenerative medicine has been developing as a medical treatment for intractable diseases to replace the transplantation of organs. Regenerative treatment procedures have already been applied to the epidermis [1, 2], cartilage [3], and retina [4]. Moreover, transplantation of human pluripotent stem cell-derived cardiomyocytes into the infarcted hearts of immunodeficient mice was recently proven to improve cardiac function [5]. However, treatment of cardiac insufficiency in humans can require as many as 10^9^ cardiomyocytes differentiated from human induced pluripotent stem cells [6]. Therefore, mass culture of cells, in which engineers should play a substantial role, is one of the most important requirements for dissemination of regenerative medicine, and various efforts have been made toward the improvement of cell culture processes to date.

Efficiency of cell proliferation is one of the main issues for increasing the scale of cultures. Because the cell cycle itself cannot be dramatically accelerated, preparation of an appropriate culture environment is important for effective cell development. However, culture operations are generally conducted manually by technicians with different levels of experience/skill and involve immense amounts of labor, leading to wide variability. Therefore, for efficient cell proliferation, it is necessary to realize automated and uniform cell culture techniques that are not dependent on manual procedures.

For cell culture processes excluding cell proliferation (i.e. cell seeding, detachment, and collection), methods that do not require manual procedures have been reported. The reported detachment methods have included a temperature-responsive polymer-coated culture dish [7, 8] and a cell culture device employing a piezoelectric ultrasonic transducer [9–11]. For cell collection, ultrasonic pumping has been introduced to collect the cell suspension [12]. However, even though the initial cell distribution has a large effect on cell proliferation, there are very few reports on efficient cell seeding methods. The reason why the cell distribution is important is that dense cell regions can decrease cell development, an effect known as contact inhibition [13]. Development of an effective method to homogenize the initial cell distribution may contribute to improved efficiency of cell proliferation.

Therefore, the present study aimed to develop a method for homogenization of the cell seeding density using secondary flow induced by orbital movement of the culture dish. A speed-controllable orbital shaker was developed, and the efficiency of the proposed method was evaluated.

## Theory

It is possible to generate a swirl flow of medium in a circular culture dish by rotating the dish along a circular orbit. At this time, the Ekman layer expressed as

$$\varepsilon =\sqrt{\frac{\nu }{\omega }}$$ (1)

is generated in the medium, where *ε*, *ν*, and *ω* represent the Ekman layer thickness, kinematic viscosity, and angular velocity, respectively [14],[15]. The Ekman layer is a viscous layer near the bottom surface, in which the centrifugal force applied to particles is decreased because the viscous drag reduces the swirl flow velocity. At the same time, owing to the rotation of the dish along a circular orbit, sloshing is induced on the surface of the medium, and the medium height becomes higher at the outer circumference than in the center [16]. Consequently, a constant centripetal force caused by the pressure gradient appears in the Ekman layer, inducing a secondary flow. The constant centripetal force due to the pressure gradient depends on the angular frequency of lateral sloshing. The primary resonant angular frequency of lateral sloshing, *ω*, is expressed as

$$\omega ={\left[g\frac{2.83}{R}\text{tanh}\left(2.83\frac{h}{R}\right)\right]}^{\frac{1}{2}}$$ (2)

where *g*, *R*, and *h* represent the gravity, vessel radius, and liquid level, respectively [16]. As a result, particles move toward the center of the circular dish [17], and the movement is designated Ekman transportation.

**Fig 1:**
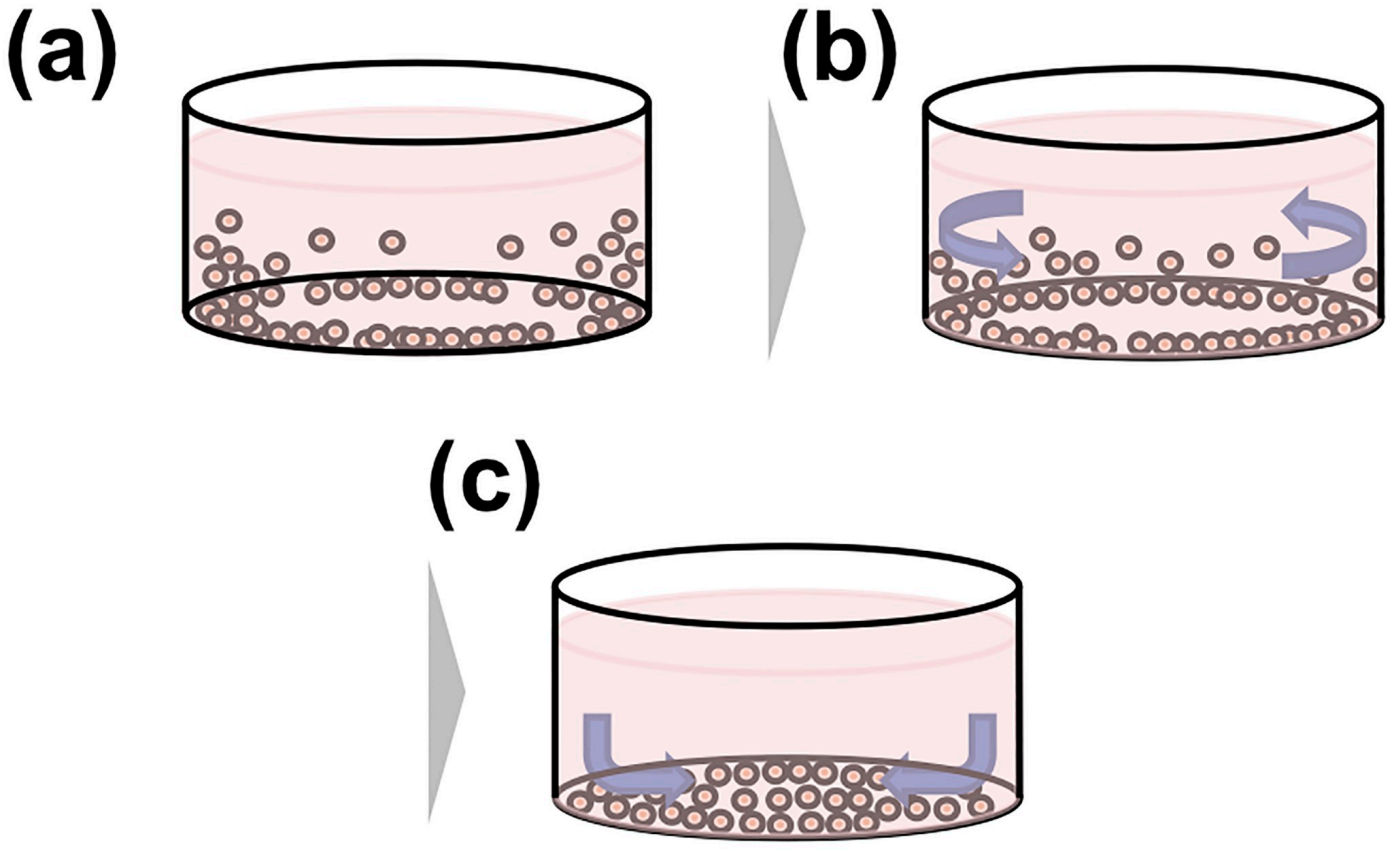
Concept of the cell density homogenization method using Ekman transportation. (a) Schematic diagram of the initial cell distribution after seeding with a pipette. The cell density around the edge of the dish is higher than that at the center of the dish. (b) A swirl flow is induced by rotating the dish along a circular orbit. (c) Due to the swirl flow, a secondary flow directed toward the center is induced in the cell suspension. The cells accumulated around the edge are moved to the center of the dish.

The concept for homogenizing the distribution of seeded cells based on Ekman transportation is shown in Fig 1. In general, cells seeded in a dish through a pipette gather around the outer wall of the dish due to the pipetting pressure and the surface tension against the wall. Notably, such high-density regions of cells can decrease the proliferation [18]. Thus, a swirl flow is induced in the cell suspension by shaking the dish along a circular orbit. This swirl flow induces a secondary flow, and the cells located near the wall are moved toward the center of the dish.

## Materials and methods

### Measurement of rotational speed of an orbital shaker device

The relationship between the rotational speed of an orbital shaker device (OSD) and the applied voltage was measured by a tachometer (HT-5500; Ono Sokki, Kanagawa, Japan) in a noncontact manner while applying voltages of 3–12 V.

### Preparation of cells

The mouse myoblast cell line C2C12 (RCB0987; Riken Bio Resource Center, Ibaraki, Japan) was employed as a representative adherent cell line for cell seeding experiments, because C2C12 cells have been used for studies of maturation related to cell density [19] and cell patterning [20]. After thawing, the cells were cultured in the 75 cm^2^ flasks (90075, TPP Techno Plastic Products AG, Trasadingen, Switzerland) containing culture medium (DMEM with phenol red) supplemented with 10% fetal bovine serum in a 5% CO_2_ humidified atmosphere incubator at 37 °C until they reached 80% confluence. To detach the cells from the flask, the cells were immersed in 0.05% trypsin-EDTA (25300; Life Technologies, Carlsbad, CA, USA) for 3 min [21]. The detached cells were collected and a quarter of the cells were seeded into other flasks for subculture. Subsequently, 5.7 × 10^5^ cells were seeded into 35-mm dishes (150460, Nalge Nunc International, NY, USA) with 2 mL of serum-free medium. Calcein (Calcein-AM Solution; Doujinkagaku Laboratory Corporation, Kumamoto, Japan) was added to the samples for staining the viable cells. Since calcein stains the cytoplasm of viable cells in response to calcium, serum-free medium without calcium was used.

### Homogenization of cell distribution by orbital shaking

The cells in each dish were cultured for 3 min while shaking on an OSD at 35, 61, or 87 rpm in the incubator. A sample without shaking was prepared as the control. Next, to facilitate cell adherence to the culture surface and achieve effective staining, the samples were left to stand in the incubator for 40 min.

### Measurement of cell distribution

**Fig 2:**
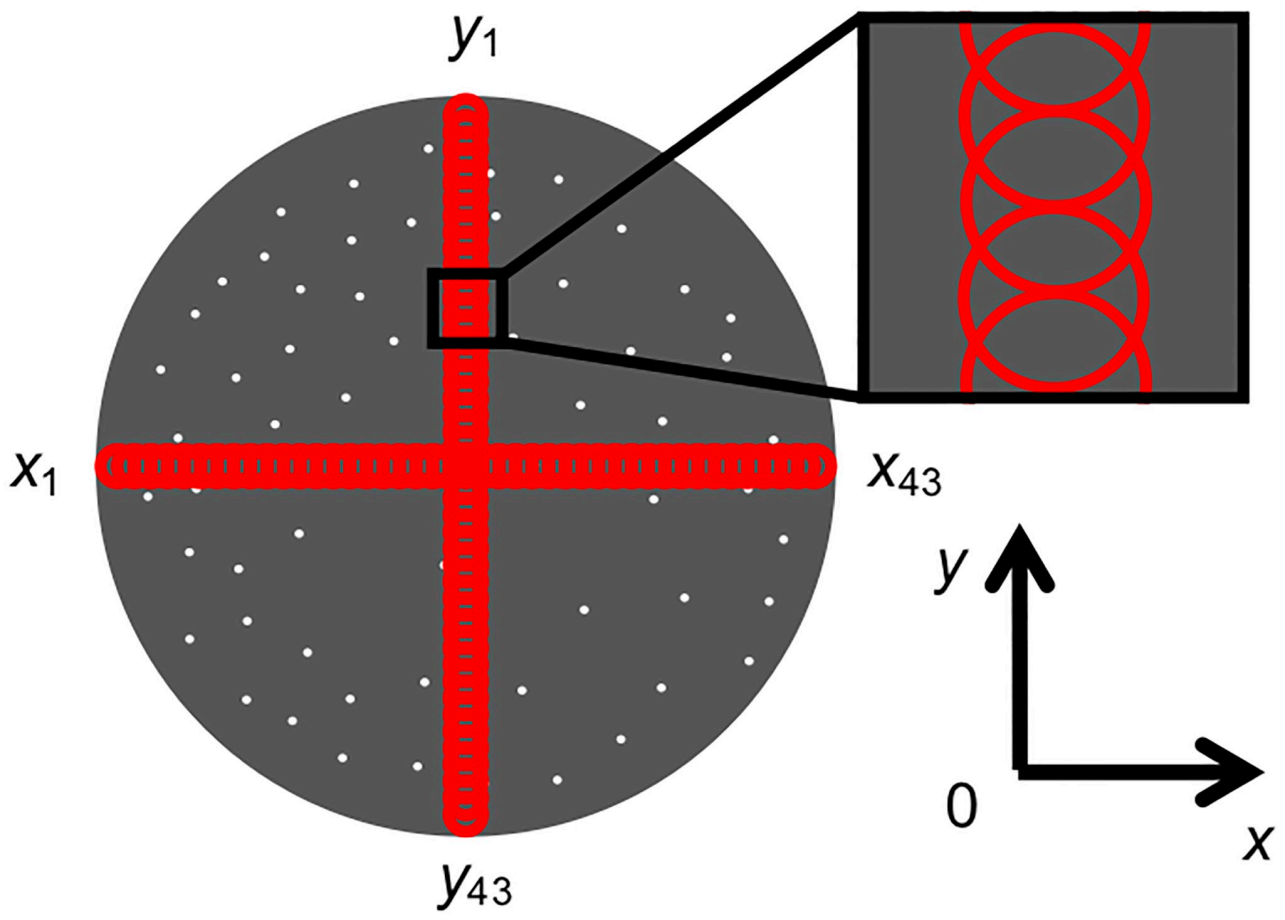
Measurement areas for evaluation of cell distribution.

For evaluation of local cell density, the cell distribution on the culture surface was measured. A fluorescence image of the entire culture surface was obtained using a phase-contrast and fluorescence microscope (ECLIPSE; Nikon, Tokyo, Japan). The obtained image was binarized by Image J software (National Institutes of Health, Bethesda, MD, USA). As shown in Fig 2, we defined 43 circles with a diameter of 1.4 mm at equal intervals along the diametrical directions of the culture surface (*x* and *y* directions), and obtained the area occupied by cells in each circle.

### Measurement of kinematic viscosity

To calculate the Ekman layer thickness (c.f. (1)), the viscosity is measured and the kinematic viscosity, *v*, was calculated as

$$\nu =\frac{\mu }{\rho }$$ (3)

where *μ* and *ρ* represent the viscosity and density, respectively. Note that, the density of the medium was measured by averaging the mass of 1000 μL of the medium using an electric balance (GR-200, Yamato Scientific Co., Ltd., Tokyo, Japan) at 8 times. The viscosity was measured by a viscometer (DV1 Digital Viscometer; Brookfield Ametek, Middleborough, MA, USA). Serum-free medium at 37 °C was added to a container of about 30 mL and its viscosity was measured for 50 s.

### Evaluation of cell proliferation after orbital shaking

To confirm the cell proliferation after stirring by the OSD, the numbers of cultured cells at 24 h after homogenization at the best rotational speed (61 rpm) and control cells were counted. For this, 5.7 × 10^5^ cells were seeded in 35-mm dishes with 2 mL of culture medium (without fetal bovine serum) and placed in the 5% CO_2_ humidified atmosphere incubator at 37 °C, wherein four samples were shaken by the OSD for 3 min at 61 rpm. On the other hand, four non-stirred control samples were left stationary for 3 min in the incubator. All samples were then cultured for 24 h in the incubator. After 24 h, the numbers of cells were counted. Briefly, the cells were immersed in 0.05% trypsin-EDTA for 4 min, detached from the dish, and counted with a cell counter (TC20^™^ Automated Cell Counter; Bio-Rad, Hercules, CA, USA).

### Statistical analysis

Statistical analyses were performed by analysis of variance (ANOVA). The statistical significance of differences in data by ANOVA was evaluated by SPSS software (IBM SPSS Statistics V25; IBM Corp., New York, NY, USA) with the Tukey method. Values of *p* < 0.05 or *p* < 0.01 were accepted as statistically significant.

## Results

### Fabrication of the OSD

**Fig 3:**
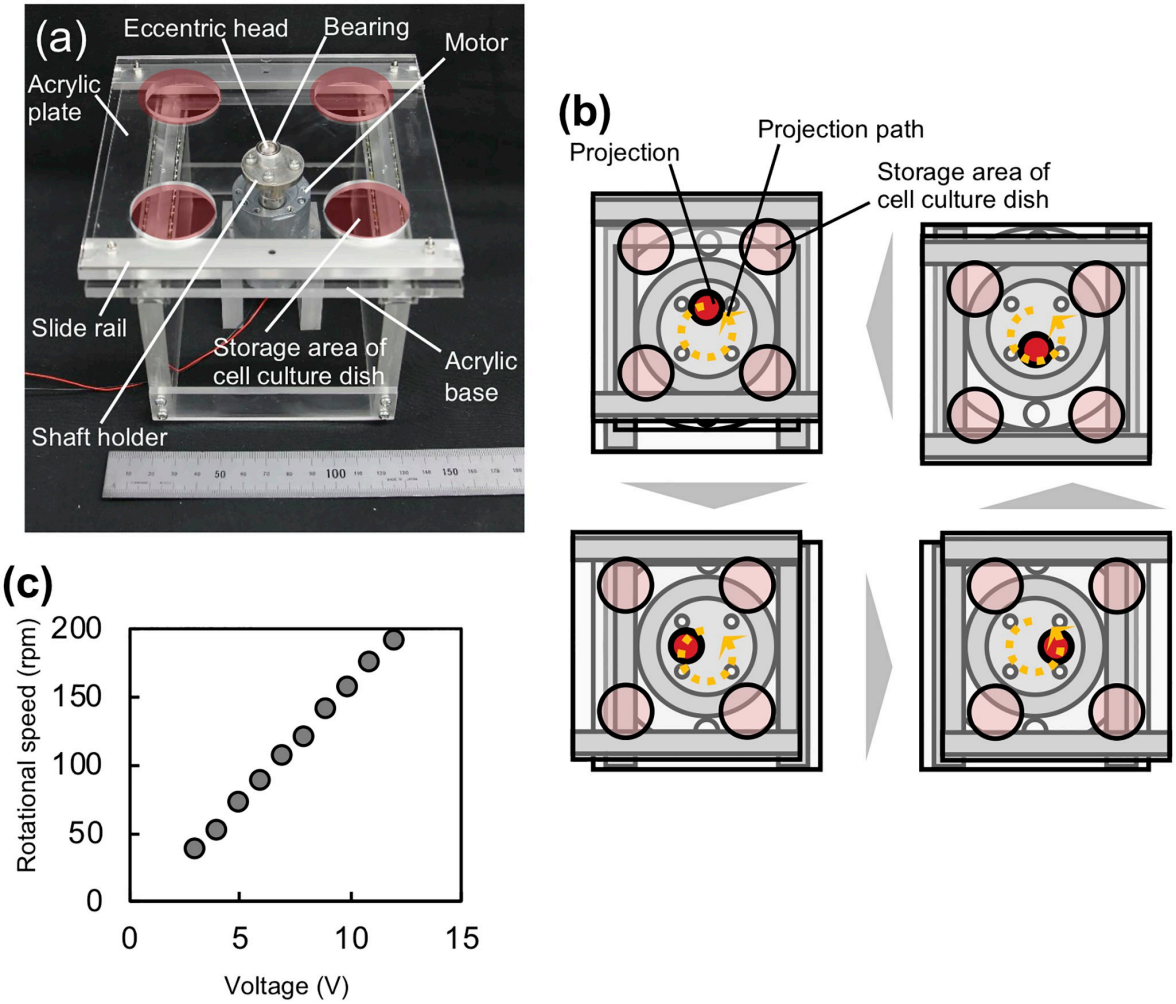
Details of the orbital shaking device to homogenize the initial cell density in 35-mm cell culture dishes. (a) Overall view of the device. (b) Motion of the device. (c) Relationship between the input voltage and the rotational speed.

The OSD is shown in Fig 3a. The OSD comprises *x* and *y* slide rails, a motor, an eccentric head, a bearing, an acrylic base, and an acrylic plate. The OSD contains four holes (denoted by red circles) in the acrylic plate for mounting consumable 35-mm dishes. The eccentric head is mounted on the motor and has a projection located at 2 mm from the center. The projection is inserted into a small hole located at the center of the acrylic plate. By driving the motor, the acrylic plate moves eccentrically along a circular orbit drawn by the projection (Fig 3b). Consequently, the dishes move on a horizontal plane along a circular trajectory with a radius of 2 mm. Rotational speed of the motor can be adjusted by applying different input voltages. As shown in Fig 3c, the rotational speed was proportional to the input voltage.

### Variation in cell density distribution by orbital shaking

**Fig 4:**
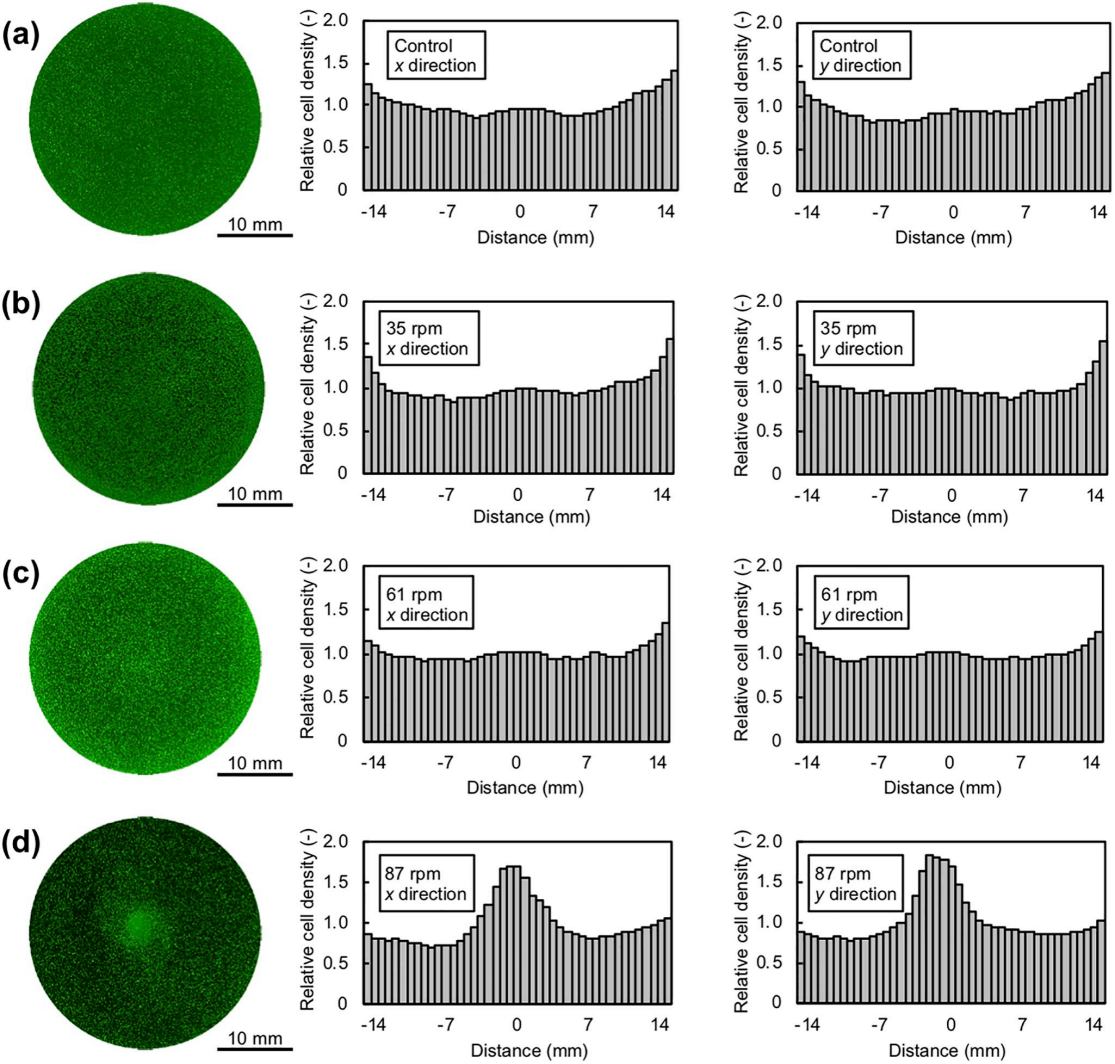
Fluorescence microscopy images of culture dishes and relationship between relative cell densities along the *x* and *y* directions (*n* = 3). (a-d) Cells were seeded in culture dishes without shaking (a) or with shaking at 35 rpm (b), 61 rpm (c), or 87 rpm (d) for 3 min. The green fluorescence shows viable cells.

**Fig 5:**
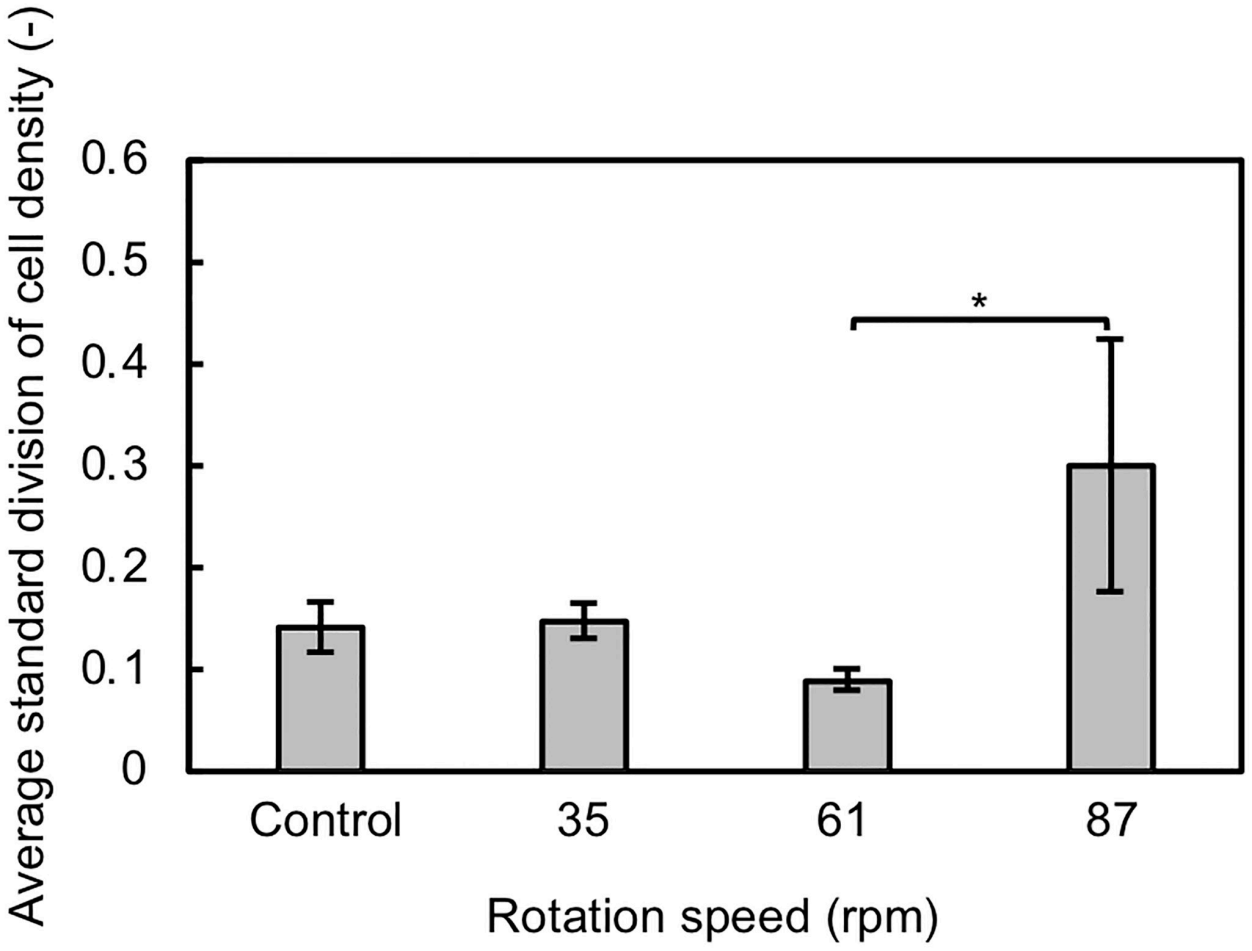
Standard deviation of cell density for samples at each rotation speed. Data are shown as mean ± SD (*n* = 3). *p < 0.05.

Fig 4 shows the fluorescence microscopy images and relative distributions of cells. The distribution was determined by the relationship between the relative cell density and the distance from the dish center. The fluorescence microscopy images of the cells were binarized, and the cell density was calculated as the proportion of cells within each circular area defined in Fig 2. The cell density was then divided by the mean cell density of every area shown in Fig 4 to obtain the relative distribution. Under all conditions, the cell distribution was almost symmetrical relative to the center of the dish, and no different features were observed along the *x* and *y* directions. These results indicated that the cell distribution was axisymmetric at any position. As shown in Fig 4a, the cell density in the control sample was higher near the dish wall than in the center of the dish. In other words, inhomogeneity of the cell distribution occurred in the manual cell seeding process with a pipette. After stirring with the OSD, the cell distribution at low rotational speed (35 rpm; Fig 4b) was similar to the control, while that at moderate rotational speed (61 rpm; Fig 4c) was much more homogeneous, and that at high rotational speed (87 rpm; Fig 4d) showed higher density at the center of the dish. To quantitatively evaluate the homogeneity of the cell distribution under each condition, the standard deviation of the cell density of each area shown in Fig 4 was shown in Fig 5. The results indicated that the distribution of cells in the culture dish was adjusted by the rotational speed of the OSD, and that the dispersion of the initial cell distribution was relaxed.

### Ekman layer thickness and angular frequency of lateral sloshing

The viscosity and density were measured as 1.35 mPa·s and 1009.73 kg/m^3^, respectively. Thus from (3), the kinematic viscosity was calculated as 1.34 × 10^−6^ m^2^/s. Therefore, the Ekman layer thicknesses were 605 μm (35 rpm), 458 μm (61 rpm), and 384 μm (87 rpm) based on calculations with (1).

From the calculation of the angular frequency of lateral sloshing (c.f. (2)), the resonant angular frequency of lateral sloshing of a medium is calculated as 9.11 rad/s (= 87.0 rpm). This means that, among the range of angular frequencies used in this study, the faster the angular velocity was, the higher the wave height was, i.e. the higher the pressure gradient became. As a result, when the rotational speed was higher, the cells received larger force and the cell density at the center of the culture surface was increased.

### Cell proliferation with different initial cell distributions

**Fig 6:**
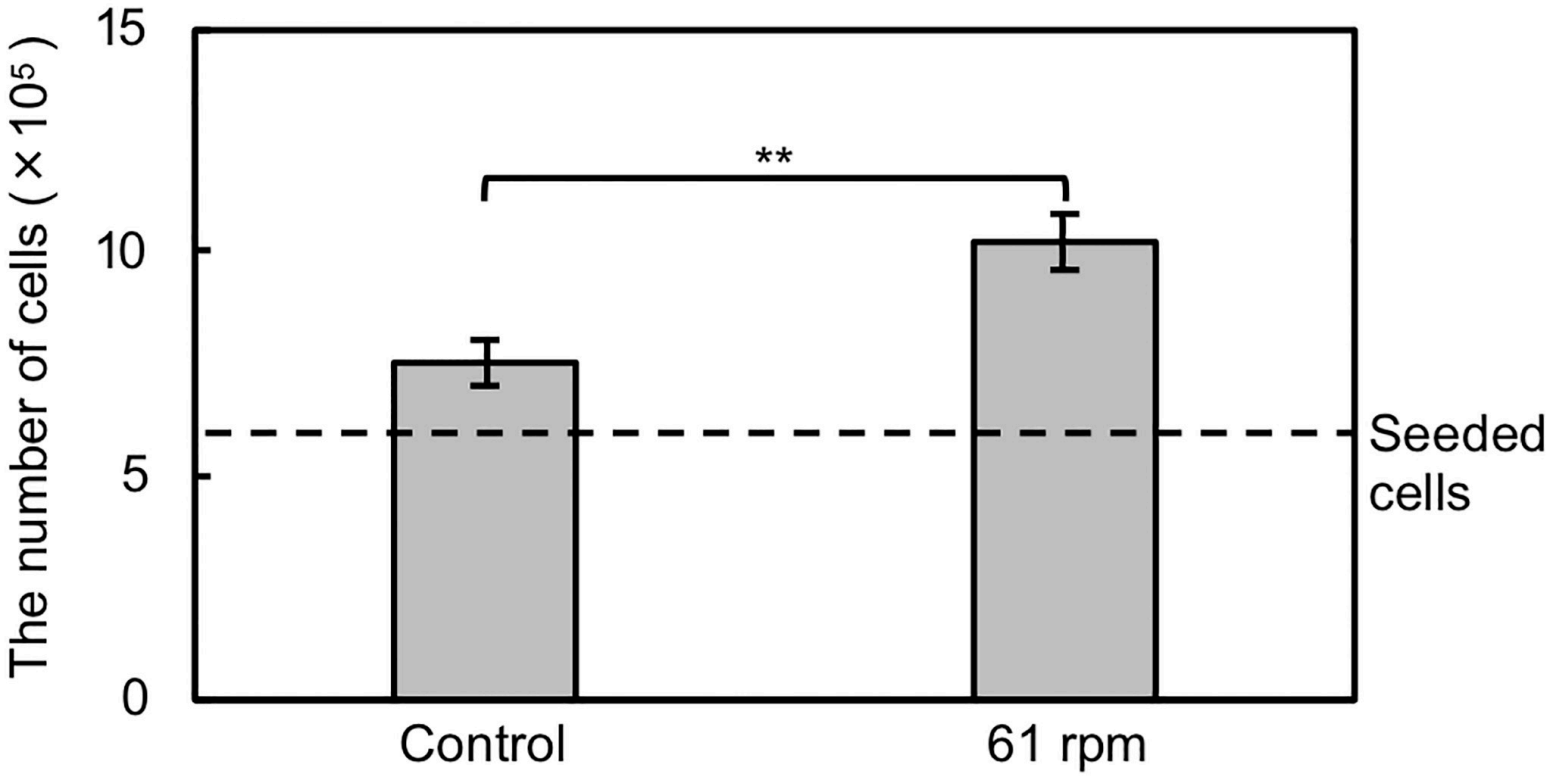
Numbers of cells after 24 h of culture. The initial cell density was homogenized by orbital shaking at 61 rpm. Data are shown as mean ± SD (*n* = 4). **p < 0.01.

The cell proliferation after homogenization of the initial cell density was evaluated. The numbers of cultured cells at 24 h after the initial cell density was homogenized at moderate rotation speed (61 rpm) are shown in Fig 6. The number of cells stirred at moderate rotation speed was 36% higher than that of control cells with a significant difference (*p* < 0.01, by ANOVA). This result indicates that homogenization of the initial cell density by Ekman transportation contributed to the achievement of higher cell proliferation.

## Discussion

The purpose of this study was to homogenize the density of cells initially seeded into a culture dish using an orbital shaker. In general, the distribution of the seeded cells was inhomogeneous, and the cells were more likely to aggregate around the wall of the culture dish (Fig 4a). As factors for this inhomogeneous distribution, warpage of the cell culture dish and meniscus formation along the wall of the dish are possible candidates. Standard culture dishes are made of polystyrene and manufactured by injection molding. Injection molding induces warpage of plastic parts due to inhomogeneous shrinkage of the material [22]. This warpage of a cell culture dish, which is high at the center of the dish, can easily be observed with a bubble level vial (data not shown). In addition, a meniscus, defined as the curve appearing in the upper surface of a liquid, is formed between the dish wall and the medium due to surface tension. The meniscus causes convection due to evaporation from the medium. In particular, convection occurs along one direction to compensate for the medium at the edge [23–25]by evaporation of the medium around the meniscus. Hence, the warpage and the convection due to meniscus formation both induce outward movement of cells in the culture dish.

By employing Ekman transportation, the cells were more likely to aggregate at the center of the culture dish with increasing rotational speed, as shown in Fig 4b–4d. As the rotational speed increased, the centripetal force acting on the cells increased. In other words, among the three rotation speeds (35, 61, and 87 rpm), the rotation speed of 87 rpm coincides with the sloshing of (1.1) mode, so that the constant centripetal force increases as rotation speed approaches to 87 rpm. Once medium sloshing occurred because of the orbital rotation of the dish [26] and the centripetal force due to the pressure gradient acting on the cells became significant, the cells moved toward the center of the dish. Under the experimental conditions employed in the present study, the Ekman layer at each speed (35, 61, and 87 rpm) was sufficiently thicker (~100 μm) than the cell height (~3 μm) [27], meaning that the cells were subjected to the centripetal force and moved toward the center of the dish.

Cultured cells exchange signals with one another. When the distribution of seeded cells is inhomogeneous, a local high-density area of seeded cells occurs and the proliferation is decreased by contact inhibition [28, 29]. Thus, cell proliferation must be most effectively improved by seeding cells at equal intervals. In our research, proliferation of cells after shaking at moderate rotation speed (61 rpm) was significantly larger than that of control cells. This may be due to the homogeneous distribution of cells, as confirmed in Figs 4 and 5.

For bioengineering applications such as regenerative medicine, disposable standard culture dishes are preferred to avoid contamination and cross-contamination. Our method is able to homogenize the distribution of initially seeded cells using standard dishes, meaning that it is highly compatible with medical applications. Cell culture efficiency can be dramatically improved by the proposed method, as confirmed in Fig 6.

## Conclusions

We have developed a method to homogenize the initially seeded cell density in a standard culture dish by using Ekman transportation induced by an orbital shaker. Shaking the culture medium in a 35-mm dish at 61 rpm with an eccentric distance of 2 mm led to homogeneous cell density compared with the conventional seeding method using a pipette (control). The proliferation of cells shaken by the proposed method was significantly higher than that of control cells because the shaken cells were more equally seeded on the culture surface. This cell density homogenization method can provide a fundamental technique for efficient cell culture.

## Supporting information

S1 File Data set is provided as a S1 File. (XLSX)
